# A fat suppressed adiabatic T2-preparation module for 3T

**DOI:** 10.1186/1532-429X-15-S1-E52

**Published:** 2013-01-30

**Authors:** Panki Kim, David Wendell, Eun-Ah Park, Hyeonjin Kim, Whal Lee, Wolfgang G Rehwald

**Affiliations:** 1 Radiology, Seoul National University Hospital, Seoul, Republic of Korea; 2 Cardiology, Duke University, Durham, NC, USA; 3 Siemens Healthcare Cardiac MR R&D, Chicago, IL, USA

## Background

A recently developed T2-preparation module for cardiac imaging at high field is insensitive to B0 and B1 inhomogeneity, cardiac motion and flow. Obtained T2 weighted images exhibit bright fat signal that can obscure the signal of myocardium and other muscles. Therefore, a chemical selective saturation (CHESS) is usually applied for fat suppression after the T2-preparation, e.g. in coronary artery MRI. Its quality is often poor and it is unsuited for longer readout durations. In this study, we propose a new fat suppressed T2-preparation module for 3T using binomial composite RF pulses as 90° pulses to more efficiently suppress fat, maintaining robustness towards inhomogeneity, cardiac motion, and flow.

## Methods

**Figure 1:**
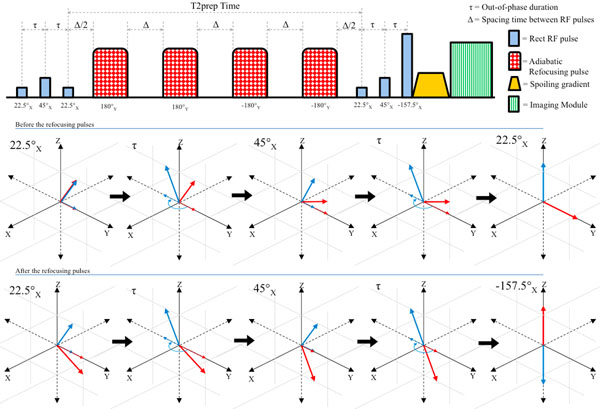
Sequence diagram for proposed fat-suppressed adiabatic T2-preparation pulse. This preparation module is composed of three parts: 1) a composite binomial pulse consisting of 3 rectangular pulses with 1:2:1 amplitude ratio for only selecting water spins, 2) four adiabatic refocusing pulses (BIREF-1), and 3) a composite binomial pulse consisting of 3 rectangular pulses with 1:2:7 amplitude ratio to arrange water and fat spins on the +Z and -Z axis, respectively.

An oil and water phantom was scanned on a Siemens MAGNETOM Trio 3T MR scanner (16-channel coil) to compare the proposed fat-suppression method with the existing T2-preparation using CHESS fat-suppression.The proposed T2-preparation method as shown figure 1 with integrated fat-suppression uses refocusing pulses shown in a previous study [1] to be insensitive to cardiac motion, blood flow, B0 and B1 field. The preparation module is composed of 3 parts. First, a composite binomial pulse consisting of 3 rectangular pulses with1:2:1 amplitude ratio for tipping water spins onto the transverse plane using 22.5° - 45° - 22.5° flip angles. Second, four BIREF-1 [2] pulses for refocusing water spins. Third, a composite binomial pulse (22.5° - 45° - -157.5°) to rotate water and fat spins onto the +Z and -Z axis, respectively. Fat suppression was achieved when the inverted fat spins reached the zero crossing of the fat recovery curve while acquiring the center of k-space during segmented scanning. The scan parameters were ECG gated 2D FLASH sequence, TR/TE = 4.04/1.63 ms, T2-prep time = 40 ms, # of segments = 21, water-fat out of phase time = 1.23 ms, flip angle = 20, FOV = 250 x 210, Matrix = 256 x 216, Thickness = 5 mm, simulation RR = 800 ms, trigger pulse = 2, trigger time = 580 ms. To evaluate the fat suppression capability of the proposed method, signal-to-noise ratio (SNR), water-fat signal ratio and fat heterogeneity were measured. The heterogeneity was defined as standard deviation of normalized fat signal intensity.

## Results

**Figure 2:**
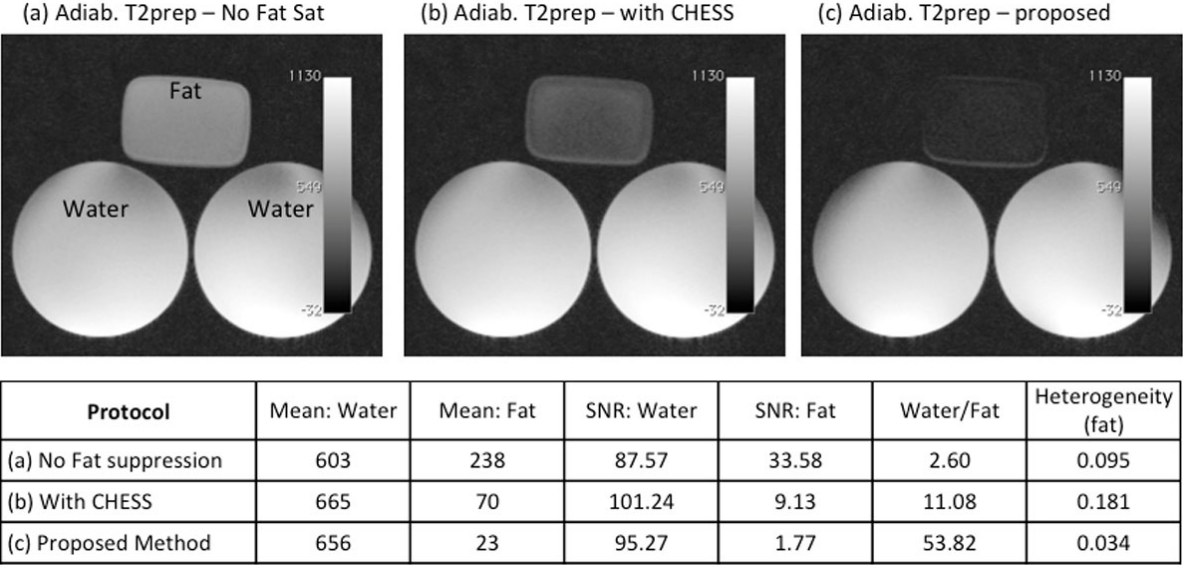
Images obtained with (a) the adiabatic T2prep module without any fat suppression, (b) the adiabatic T2prep with CHESS pulse, and (c) the proposed T2prep module with integrated fat-suppression. Fat signal was significantly and uniformly suppressed by the proposed method.

Figure 2 shows the images acquired with the adiabatic T2-preparation and with different fat suppression techniques. Figure 2(a) shows an image generated by the adiabatic T2-preparation pulse without any fat-suppression, (b) by adiabatic T2-preparation pulse with existing CHESS RF pulse for fat suppression, and (c) by our proposed method. These images have identical window level. Also, the proposed method has a higher water/fat ratio (53.82) and lower fat heterogeneity (0.034) than the existing method, see table below.

## Conclusions

The proposed T2-preparation method with integrated fat-suppression has superior fat suppression performance than existing techniques.

## Funding

This research was supported by Leading Foreign Research Institute Recruitment Program through the National Research Foundation of Korea (NRF) funded by the Ministry of Education, Science and Technology (MEST) (K21002001824-11E0100-02910).

